# Insulin Autoimmune Syndrome (Hirata’s Disease) Caused by Methimazole in a 47-Year-Old Man

**DOI:** 10.7759/cureus.68146

**Published:** 2024-08-29

**Authors:** Lisa A Abraham, Sualeha Khalid, Abraham Halik, Rana Malek

**Affiliations:** 1 Internal Medicine, University of Maryland Medical Center, Baltimore, USA; 2 Endocrinology, University of Maryland Medical Center, Baltimore, USA; 3 Endocrinology, Diabetes and Metabolism, University of Maryland School of Medicine, Baltimore, USA

**Keywords:** insulin antibody assessment, hyperinsulinemic hypoglycemia, insulin autoantibodies, methimazole, insulin autoimmune syndrome

## Abstract

Insulin autoimmune syndrome (IAS), or Hirata’s disease, is a rare disease characterized by episodes of hypoglycemia with elevated levels of insulin secondary to high concentrations of insulin autoantibodies. The use of methimazole is a risk factor for the development of Hirata’s disease. We report a case of a 47-year-old man being treated for thyroid storm initially with methimazole and other agents. Medical management was stopped, as the patient was refractory to treatment. Ultimately, the patient underwent plasmapheresis and thyroidectomy. While the patient was initially noted to have hyperglycemia during his hospital stay, requiring a regular insulin drip, he subsequently developed hypoglycemia even after cessation of insulin therapy. Lab work was positive for insulin autoantibodies, and the patient was diagnosed with IAS. IAS should be considered in patients with hypoglycemia who have been exposed to specific agents.

## Introduction

Insulin autoimmune syndrome (IAS), or Hirata’s disease, is a rare disease characterized by episodes of hypoglycemia with elevated levels of insulin secondary to high concentrations of insulin autoantibodies [1]. The incidence of IAS is still unknown, most likely due to difficulties in diagnostic workup and unawareness of the disease until the last decade [1]. Based on a summary of the main epidemiological studies on IAS, 380 cases were reported worldwide, from 1970 to 2009 [1]. Several medications have been identified as triggers for IAS, including methimazole [2]. Drugs containing sulfhydryl groups, such as lipoic acid, are associated with IAS, along with some other drugs such as clopidogrel, proton pump inhibitors, antituberculosis drugs, and albumin preparations [3]. Symptoms of IAS include hypoglycemia and episodes of hypoglycemia and hyperglycemia, which include hunger, tremor, anxiety, confusion, and more severely, loss of consciousness [1,3]. The Whipple triad can be used as the first step in diagnosis, and this includes symptoms of hypoglycemia, recorded low plasma glucose concentrations, and relief of symptoms after administration of glucose [1,3]. IAS should be differentiated from other causes of hyperinsulinemic hypoglycemia such as insulinoma, hypoglycemia from insulin receptor antibodies, and hypoglycemia from insulin and its analogs [1,3]. Measurement of serum insulin concentrations during a hypoglycemic episode can help distinguish IAS from insulinoma and other causes, as insulin concentrations are usually above 1000 mU/L [1,3]. The presence of insulin autoantibodies is necessary for the diagnosis of IAS [1]. IAS appears to be a self-remitting disease, with episodes of hypoglycemia decreasing over time with antibodies decreasing over time [3]. The primary treatment for IAS and decreasing hypoglycemic episodes includes discontinuing IAS-related drugs, eating small meals, and avoiding monosaccharides, with good outcomes [3]. Other proposed treatments that could be used when first-line treatments are not effective include alpha-glucosidase inhibitors, glucocorticoids, immunosuppressants, plasma exchange, and monoclonal antibodies [3]. Here, we present a case of IAS in a patient treated with methimazole for thyrotoxicosis.

## Case presentation

A 47-year-old man was found unresponsive at home and was presented to the emergency department. He was noted to have a past medical history of heart failure with reduced ejection fraction, atrial fibrillation, hypertension, and pulmonary embolism. He did not have any known past surgical history. Home medication fill history included amiodarone 200 mg once daily, furosemide 40 mg once daily, metoprolol succinate 200 mg once daily, losartan 100 mg once daily, and spironolactone 25 mg once daily. No anticoagulants were noted in his home medications. He was noted to be a former smoker with occasional alcohol use. Vitals on arrival showed a blood pressure of 138/114 mmHg, pulse of 130 beats per minute, temperature of 36.7 degrees Celsius, respiratory rate of 21 breaths per minute, and an oxygen saturation of 96%. Physical exam findings were consistent with a left middle cerebral artery (MCA) stroke, including aphasia, right upper extremity pronator drift, and right lower extremity greater than left lower extremity drift.

**Figure 1:**
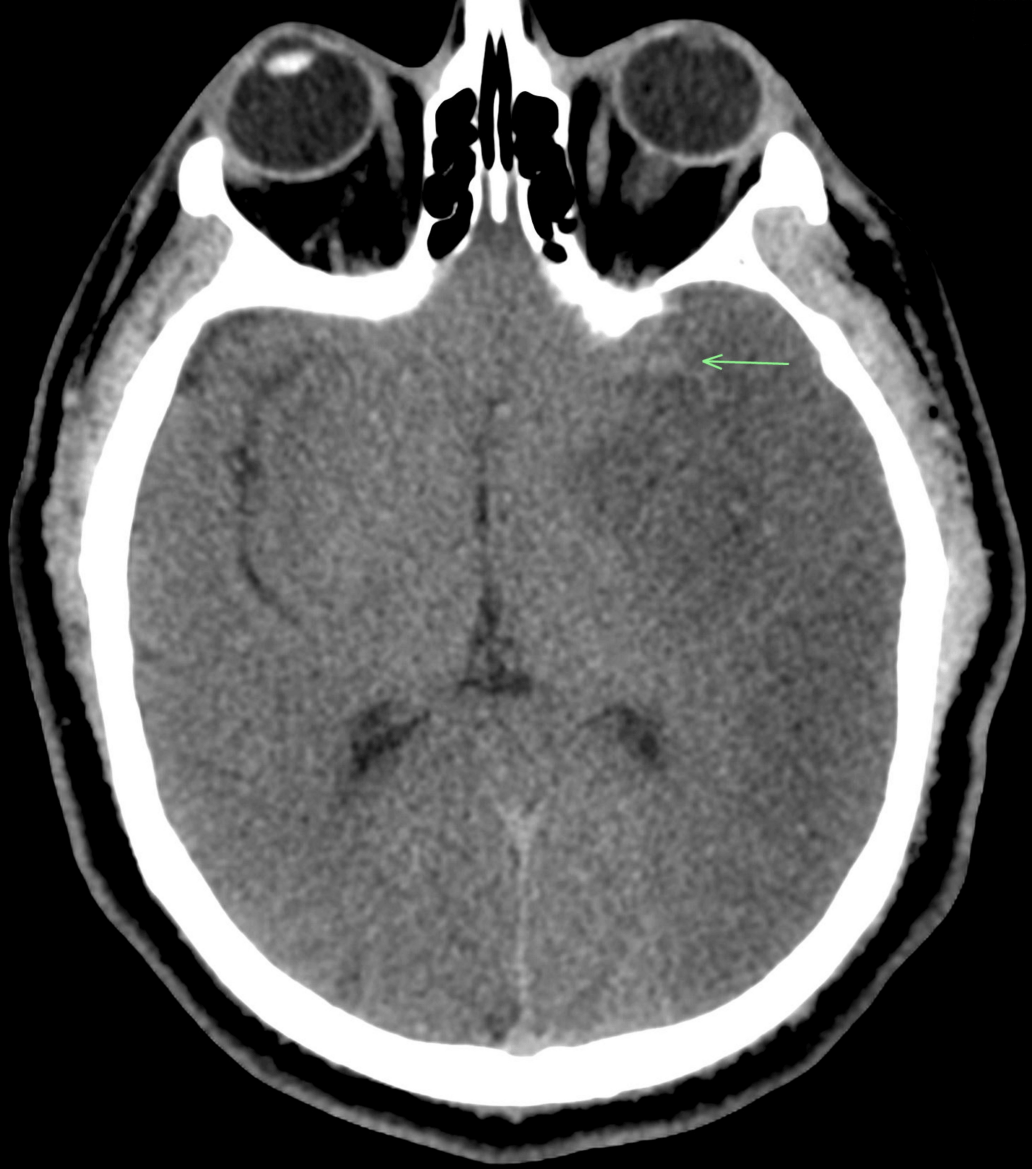
Computed tomography (CT) of the head showing left MCA occlusion and evolving infarct MCA: middle cerebral artery

**Figure 2:**
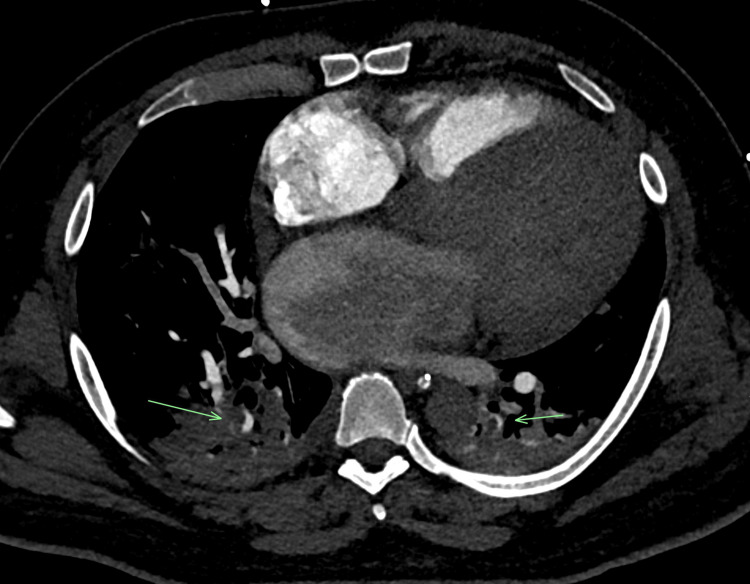
Computed tomography angiography (CTA) of the chest showing bilateral filling defects in the lungs demonstrative of bilateral pulmonary emboli

The patient was found to have a left MCA stroke due to left M1 occlusion on imaging (Figure 1) and was noted to also have bilateral extensive pulmonary emboli (Figure 2). The patient was transferred to our hospital, where he underwent a thrombectomy for the left MCA ischemic stroke. The patient subsequently had cerebral edema, which required hemicraniectomy.

His hospital course was complicated by thyroid storm, which was initially treated with methimazole, cholestyramine, hydrocortisone, and Lugol’s solution. After three weeks of medical therapy without improvement, he underwent plasmapheresis and eventual thyroidectomy. The patient developed post-surgical hypothyroidism and hypocalcemia, which were treated with levothyroxine, calcitriol, ergocalciferol, and calcium carbonate, respectively.

**Figure 3:**
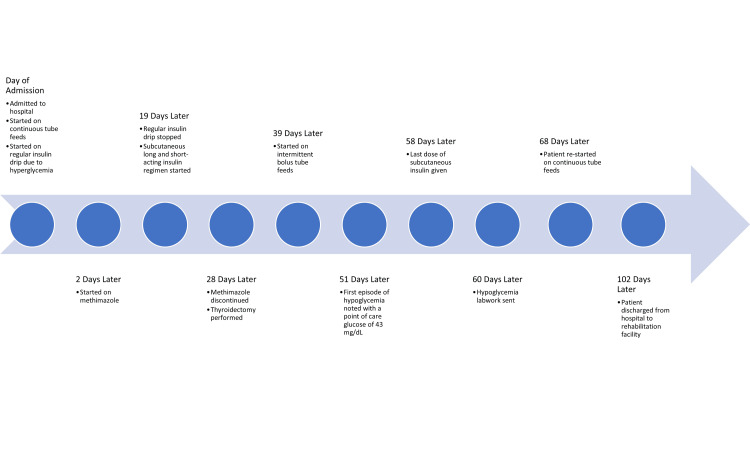
Timeline of the patient’s hospital course

On arrival at the hospital, the patient was started on tube feeds for nutrition. He was then noted to be hyperglycemic and was hence also started on a regular insulin drip the same day. Glucose levels were initially maintained at a stable range. Eventually, the patient was switched over to a subcutaneous long and short-acting insulin regimen. He was also switched over from continuous tube feeds to bolus tube feeds. Over a month into the patient’s hospital stay, the patient’s glucose levels were noted to become more and more tightly controlled until the patient had an episode of hypoglycemia, with a blood glucose of 43 mg/dL (reference range 70-99 mg/dL) in the morning. It was noted that the patient had episodes of hyperglycemia associated with his bolus tube feeds, with blood glucose levels noted to be in the 200 mg/dL (reference range 70-99 mg/dL) range. The patient would have episodes of hypoglycemia in between his tube feed boluses. Insulin glargine and short-acting sliding-scale insulin doses were decreased repeatedly until exogenous insulin was discontinued completely (Figure 3).

A hypoglycemia workup was initiated two days after receiving the last dose of insulin. Notable labs included blood glucose of 39 mg/dL (reference range 70-99 mg/dL), C-peptide of 11 ng/mL (reference range 0.5-3.3 ng/mL), insulin level of 2224 uIU/mL (reference range 3-25 uIU/mL), and beta-hydroxybutyrate of 0.6 mg/dL (reference range 0.0 -3.0 mg/dL). Given the elevated insulin levels >2000 uIU/mL and episodes of hyperglycemia and hypoglycemia off insulin therapy, insulin auto-antibody labs were sent. Elevated insulin autoantibodies > 50 U/mL (reference range 0.0-0.4 U/mL) were noted, diagnostic of insulin autoimmune syndrome. Quantification of the antibody level was not able to be performed. The patient's blood sugars continued to be monitored, and glucose levels were noted to normalize with point-of-care glucose checks performed two to three times daily. IAS was thought to be in the setting of methimazole exposure. As the patient went further out from exposure, his blood glucose stabilized with antibody clearance.

## Discussion

IAS can be divided into the drug-induced and idiopathic categories [3]. Most often, IAS is caused by medications; in fact, about half of IAS patients have a history of medication use before the onset of the disease, and about 90% use drugs containing sulfhydryl groups [3]. The most common drugs causing drug-induced IAS include methimazole and lipoic acid [3]. Idiopathic IAS is known as unexplained IAS, as there is no preceding history of drug use related to IAS [3].

Patients with Graves disease, with an allelic combination of Bw62/Cw4/DR4 carrying DRB1*04:06, were found to be at higher risk for developing IAS from methimazole use [2,4]. Most cases of IAS have been reported in East Asia due to a higher incidence of the DRB1*04:06 allele compared to Europe and North America [5]. In most cases of IAS outside Asian countries, patients are identified as white, with most cases occurring in Europe and the United States [6]. In non-Asian patients, IAS is associated with rheumatological disease or underlying hematologic diseases [6]. IAS does not appear to be reported occurring as frequently in patients of Central or South American origin, making this case unique, as the patient was originally from Mexico.

The sulfhydryl group of methimazole is known to cleave the disulfide bond of the insulin molecule, leaving the linear fragment on the a-chain exposed to the DRa-DRB1*0406 on antigen-presenting cells that bind to it with high affinity, which then activates self-insulin specific T-helper cells and the formation of insulin autoantibodies [4,7].

Thiol-containing drugs, like lipoic acid, are used as nutritional supplements. Lipoic acid can increase insulin sensitivity and improve glycemic control [3]. It is also known to promote the dissociation of the S-S bond of insulin, expose the peptide to antigen-presenting cells, and stimulate T cells, causing insulin autoantibodies to be formed [3].

The pathogenesis of IAS involves the appearance of insulin autoantibodies (IAA) [1]. IAA are immunoglobulins (Ig) directed against the endogenous insulin molecule. Because of their high binding capacity, IAA can form large antigen-antibody complexes since they can bind several molecules of insulin [1]. The low affinity for insulin, however, results in a spontaneous dissociation rate, which raises unbound insulin concentrations, resulting in hypoglycemic episodes [1]. High binding capacity and low affinity are features of IAA causing IAS [1].

Insulin antibodies that develop after exposure to exogenous insulin, on the other hand, are characterized by higher affinity and lower binding capacity than IAA, resulting in smaller antigen-antibody complexes with a lower spontaneous dissociation rate, preventing them from developing significant glycemic fluctuations [1].

IAA induces IAS with a double-phase mechanism [1]. The first phase occurs when insulin normally secreted by beta-cells in response to elevated serum glucose concentrations binds to autoantibodies, preventing the insulin from exerting its action [1]. This results in low unbound insulin concentrations and transient hyperglycemia [1]. Early postprandial hyperglycemia also allows for the release of insulin molecules that are partly bound to circulating insulin-IAA complexes and partly unbound and free to act [1]. The spontaneous dissociation of insulin from the insulin-IAA complexes does not cease when plasma glucose concentrations decrease, resulting in more unbound insulin, which further causes hypoglycemia [1].

Our patient was noted to have episodes of hypoglycemia in between tube feeds, correlating with the proposed mechanism of IAS as described above. Episodes of hyperglycemia were also noted as well. The patient remained vitally stable during these periods, with correction of the hypoglycemic episodes with glucose administration. Regarding his diagnosis of IAS, the only medication that was associated with the patient’s IAS was methimazole, which was discontinued 23 days before the first episode of hypoglycemia. The patient was also on insulin, which could also cause IAS, through the development of IAA, although this is less likely and the mechanism is less clear [3]. Sometimes, insulin antibodies develop after exposure to exogenous insulin although modern insulin has low immunogenicity [1,3]. These insulin antibodies are different from IAA because they often have a higher affinity and lower binding capacity, which leads to smaller antigen-antibody complexes with a lower spontaneous dissociation rate, which means they usually do not cause large fluctuations in glucose levels [1,3]. The measurement of the IAA titer is mandatory for the diagnosis of IAS [1].

IAS is known to be a self-remitting disease. In the case of methimazole-induced IAS, discontinuation of methimazole decreases the overall production of autoantibodies. Blood glucose normalizes after resuming a diet and with the gradual decrease of autoantibodies in the circulation [8]. A study showing long-term follow-up of IAS showed that IAA levels for two cases decreased over the course of three years, after the discontinuation of methimazole causing IAS [9]. Blood glucose levels in these patients also normalized over the course of three years [9]. Our patient had fewer hypoglycemic episodes over time and was restarted on continuous tube feeds to maintain stable glucose levels.

## Conclusions

Insulin antibody syndrome (IAS) should always be considered in the differential diagnosis of a patient with hyperinsulinemic hypoglycemia especially if a known offending agent has been prescribed recently. Insulin antibody assessment is the gold standard for diagnosis and should always be sent when working up a patient for hypoglycemia. After discontinuation of the offending agents for IAS, IAA levels should decrease over time with the normalization of blood glucose levels.

## References

[1] Cappellani D, Macchia E, Falorni A, Marchetti P (2020). Insulin autoimmune syndrome (Hirata disease): a comprehensive review fifty years after its first description. Diabetes Metab Syndr Obes.

[2] Uchigata Y, Kuwata S, Tsushima T (1993). Patients with Graves' disease who developed insulin autoimmune syndrome (Hirata disease) possess HLA-Bw62/Cw4/DR4 carrying DRB1*0406. J Clin Endocrinol Metab.

[3] Lin M, Chen Y, Ning J (2023). Insulin autoimmune syndrome: a systematic review. Int J Endocrinol.

[4] Jain N, Savani M, Agarwal M, Kadaria D (2016). Methimazole-induced insulin autoimmune syndrome. Ther Adv Endocrinol Metab.

[5] Yukina M, Nuralieva N, Solovyev M, Troshina E, Vasilyev E (2020). Insulin autoimmune syndrome. Endocrinol Diabetes Metab Case Rep.

[6] Lupsa BC, Chong AY, Cochran EK, Soos MA, Semple RK, Gorden P (2009). Autoimmune forms of hypoglycemia. Medicine (Baltimore).

[7] Matsushita S, Takahashi K, Motoki M, Komoriya K, Ikagawa S, Nishimura Y (1994). Allele specificity of structural requirement for peptides bound to HLA-DRB1*0405 and -DRB1*0406 complexes: implication for the HLA-associated susceptibility to methimazole-induced insulin autoimmune syndrome. J Exp Med.

[8] Gomez Cruz MJ, Jabbar M, Saini N (2012). Severe hypoglycemia secondary to methimazole-induced insulin autoimmune syndrome in a 16 year old African-American male. Pediatr Diabetes.

[9] Zhao L, He J, Ye S (2023). Long-term follow-up after discharge witnesses a slow decline of insulin autoantibodies in patients with insulin autoimmune syndrome complicated with Grave's disease: a report of two cases. BMC Endocr Disord.

